# Physiological and metabolomic responses of adzuki bean (*Vigna angularis*) to individual and combined chilling and waterlogging stress

**DOI:** 10.3389/fpls.2025.1598648

**Published:** 2025-05-22

**Authors:** Xiaoyan Liang, Wan Li, Huan Lu, Shihong Zhao, Jidao Du, Hongtao Xiang

**Affiliations:** ^1^ College of Agriculture, Heilongjiang Bayi Agricultural University, Daqing, Heilongjiang, China; ^2^ Heilongjiang Academy of Agricultural Sciences, Harbin, Heilongjiang, China; ^3^ Suihua Branch, Heilongjiang Academy of Agricultural Machinery Sciences, Suihua, Heilongjiang, China; ^4^ National Coarse Cereals Engineering Research Center, Daqing, Heilongjiang, China

**Keywords:** adzuki bean, chilling, waterlogging, combined stress, metabolomics, physiology response

## Abstract

**Introduction:**

Climate change exacerbates combined environmental stresses, leading to significant crop losses globally.

**Methods:**

This study investigated the physiological and metabolomic responses of adzuki bean (*Vigna angularis*) leaves to individual and combined chilling-waterlogging stresses during the flowering stage.

**Results and discussion:**

Results demonstrated that both stresses significantly inhibited photosynthesis, elevated reactive oxygen species accumulation, and induced membrane lipid peroxidation. Waterlogging exhibited more severe impacts, triggering extreme ABA accumulation and plant death at 4 days post-treatment, resulting in total yield loss. Notably, combined stresses induced antagonistic effects, reducing photosynthetic decline by 14.10-32.40% and mitigating oxidative damage by 5.79-10.75% compared to waterlogging alone after 4 days. Metabolomic analysis revealed that combined stress activated more metabolic pathways than individual stress, including flavone/flavonol biosynthesis and cGMP-PKG signaling, which are critical for plant adaptation.

**Conclusion:**

This study provides valuable insights into the physiological and metabolic mechanisms underlying adzuki bean’s response to combined chilling-waterlogging stress.

## 1 Introduction

Climate change increases the frequency of extreme weather events, severely constraining crop growth and yield in agricultural production (Hasegawa et al., 2021; Rezaei et al., 2023). Projections indicate that the incidence and severity of such events will intensify over the next two decades (Heilemann et al., 2024; Li et al., 2025). Low-temperature stress includes chilling (0-15°C) and freezing (<0°C) stress, with chilling stress occurring more frequently during critical crop growth stages. For instance, soybean crops in northeastern China experience periodic chilling during flowering and podding stage (Hu et al., 2022). Chilling stress disrupts membrane integrity, induces lipid peroxidation, and triggers reactive oxygen species (ROS) over accumulation, impairing photosynthetic electron transport and reducing photosynthetic efficiency (Ding et al., 2019). Additionally, low-temperature signaling involves multiple pathways, such as calcium ion signaling and the ICE1-CBF (inducer of CBF expression 1-C-repeat binding factors) cascade. While the ICE1-CBF pathway activates cold-responsive genes, excessive stress may disrupt signaling homeostasis (Park and Jung, 2024).

Waterlogging stress arises from inadequate drainage systems submerging roots, poor soil structure impeding water infiltration, water accumulation in low-lying areas and frequent extreme rainfall in specific areas (Liu et al., 2021; Manghwar et al., 2024). In China, flood-related disasters account for approximately 20% of total agricultural losses (Liu et al., 2023). Studies on soybean, cotton, and mung bean indicate that waterlogging-induced root hypoxia impairs nutrient uptake, induces oxidative damage, and reduces photosynthetic efficiency via stomatal closure (Zhang et al., 2021; Xiang et al., 2024; Yang et al., 2024). Under hypoxia, plants accumulate toxic metabolites (e.g., ethanol, acetaldehyde) and face energy deficits, leading to premature senescence or death in severe cases (Fukao et al., 2019; Liu et al., 2020).

Although chilling and waterlogging stresses have been extensively investigated individually, their combined effects remain poorly understood. In temperate regions, chilling stress often coincides with heavy rainfall during the growing season (Thapa et al., 2023). As a major legume crop in China, adzuki bean (*Vigna angularis*) has expanded in cultivation but remains sensitive to low temperatures and waterlogging (Xiang et al., 2024). This study administered chilling, waterlogging, and their combined treatments of the adzuki bean at flowering stage. Physiological parameters, leaf metabolomic profiles, and yield-related traits were systematically analyzed to identify adaptation mechanisms to concurrent stresses. These findings reveal conserved molecular targets for breeding multi-stress-resilient adzuki bean varieties with stabilized yields under climate extremes.

## 2 Materials and methods

### 2.1 Experimental design

This experiment was conducted at the Institute of Crop Cultivation and Tillage, Heilongjiang Academy of Agricultural Sciences (34°30’ N, 119°32’ E) in 2023. The adzuki bean (*Vigna angularis*) variety used in this study was Longxiaodou (LXD). The seeds were surface-sterilized using a 5% NaClO solution for 3 minutes before being rinsed thoroughly with distilled water and then soaked in water for 12 h at 25 °C before cultivated in plastic pots (diameter×height: 30×25 cm). Prior to sowing, each pot was filled with 15.5 kg subsoil, and 200 g topsoil was covered after sowing 5 selected seeds.

When plants reached the flowering stage at the potting field, the plants were moved into the artificial climate room and set up four treatments, which were expressed as CK (control, natural conditions); W (waterlogging); C (chilling at an average temperature of 15°C); C+W (combined chilling and waterlogging). In waterlogging treatment, water submerged soil surface about 2 cm. About chilling treatment, the temperature change in one day is shown in **Supplementary Figure 1**. Each treatment was performed in triplicate. The trial lasted for a total of 4 days. The second and third top leaves were collected daily, frozen in liquid nitrogen for 30 min, and stored in an ultra-low temperature freezer (-80°C) for subsequent studies.

### 2.2 Determination of the oxidative damage index

0.5 g leaves for each treatment were homogenized in PBS buffer (0.1 M; pH 7.3) and centrifuge at 12000 rpm at 4°C for 10 min. The supernatant was taken to determine the O_2_^·-^ (superoxide anion) production rate, H_2_O_2_ (hydrogen peroxid) and MDA (lipid peroxidation product) content. The O_2_^·-^ production rate was assayed using the hydroxylamine oxidation method, and the absorbance of O_2_^·-^ was measured at 530 nm (Xiang et al., 2024). The concentration of H_2_O_2_ was measured using the method described by Guo et al. (2022). The MDA content was measured by the 2-thiobarbituric acid (TBA) method at 532 nm, 600 nm and 450 nm (Ohkawa et al., 1979).

### 2.3 Determination of the photosynthetic parameters

Photosynthetic parameters, including net photosynthetic rate (Pn), stomatal conductance (Gs), transpiration rate (Tr), and intercellular carbon dioxide concentration (Ci) were determined using LI-6400 portable photosynthetic instrument (Li-Cor 6400, Li-Cor Inc., Nebraska, USA) between 9:00 am and 11:00 am on the day of sampling, The parameters were manually set as follows: light intensity at 1000 µmol·m⁻²·s⁻¹ PPFD, CO_2_ concentration at 400 μmol·m^-2^·s^-1^ and temperature at 25°C.

### 2.4 Determination of the metabolite

Samples were retrieved from -80°C and ground in liquid nitrogen. A 100 mg sample was weighed and 1170 µL of acetonitrile-water-formic acid solution (80:19:1, v/v), 10 µL of ISMix-A, and 20 µL of ISMix-B were added, respectively. The mixture was vortexed for 60 seconds, followed by sonication in the dark at a low temperature for 25 min. The samples were then incubated at -20°C overnight. After incubation, the samples were centrifuged at 14,000 rcf for 20 min at 4°C. The supernatants (900 µL) were transferred to a 25 mg Ostro 96-well plate for positive pressure filtration. The filters were washed with 200 µL of acetonitrile-water-formic acid solution (80:19:1, v/v). The filtrates were dried under liquid nitrogen and stored at -80°C.

Targeted metabolomics was used to determine phytohormones in leaves. The absolute quantification of plant hormones was achieved by liquid chromatography-mass spectrometry (LC-MS/MS) combined with selective reaction monitoring (SRM)/multiple reaction monitoring (MRM). Untargeted metabolomics was conducted to obtain a comprehensive profile of metabolites in the leaf samples using a liquid chromatography-high-resolution mass spectrometry (LC-HRMS). The metabolite extracts were reconstituted and filtered as described for targeted metabolomics.

The chromatographic peak area and retention time were extracted for targeted metabolomics using MultiQuant 3.0.2 software. The retention time of the target metabolites was corrected using authentic standards to facilitate metabolite identification. For untargeted metabolomics, multivariate statistical analyses, including principal component analysis (PCA) and orthogonal partial least squares discriminant analysis (OPLS-DA), were performed to identify the differences in metabolic profiles among groups. The differential metabolites were selected based on variable importance in projection (VIP) values>1.0 and *p*-values<0.05 from Student’s t-test.

### 2.5 Determination of yield and yield components

At the maturity stage, 5 plants were randomly selected from each treatment to investigate the number of pods per plant, particle number per plant, and the 100-grain weight.

### 2.6 Statistical analysis

Data entry and preliminary organization were conducted using Microsoft Excel 2019 (Microsoft, Inc., Redmond WA, USA). Subsequently, analysis of variance (ANOVA) was performed using SPSS 25.0 software (SPSS Lnc., Chicago, USA). LSD (Least significant difference) multiple comparisons were employed to assess differences among treatments, with significance set at *p*<0.05.

## 3 Results

### 3.1 Physiological response of LXD leaves under chilling and waterlogging stress

#### 3.1.1 Oxidative damage

**Figure 1:**
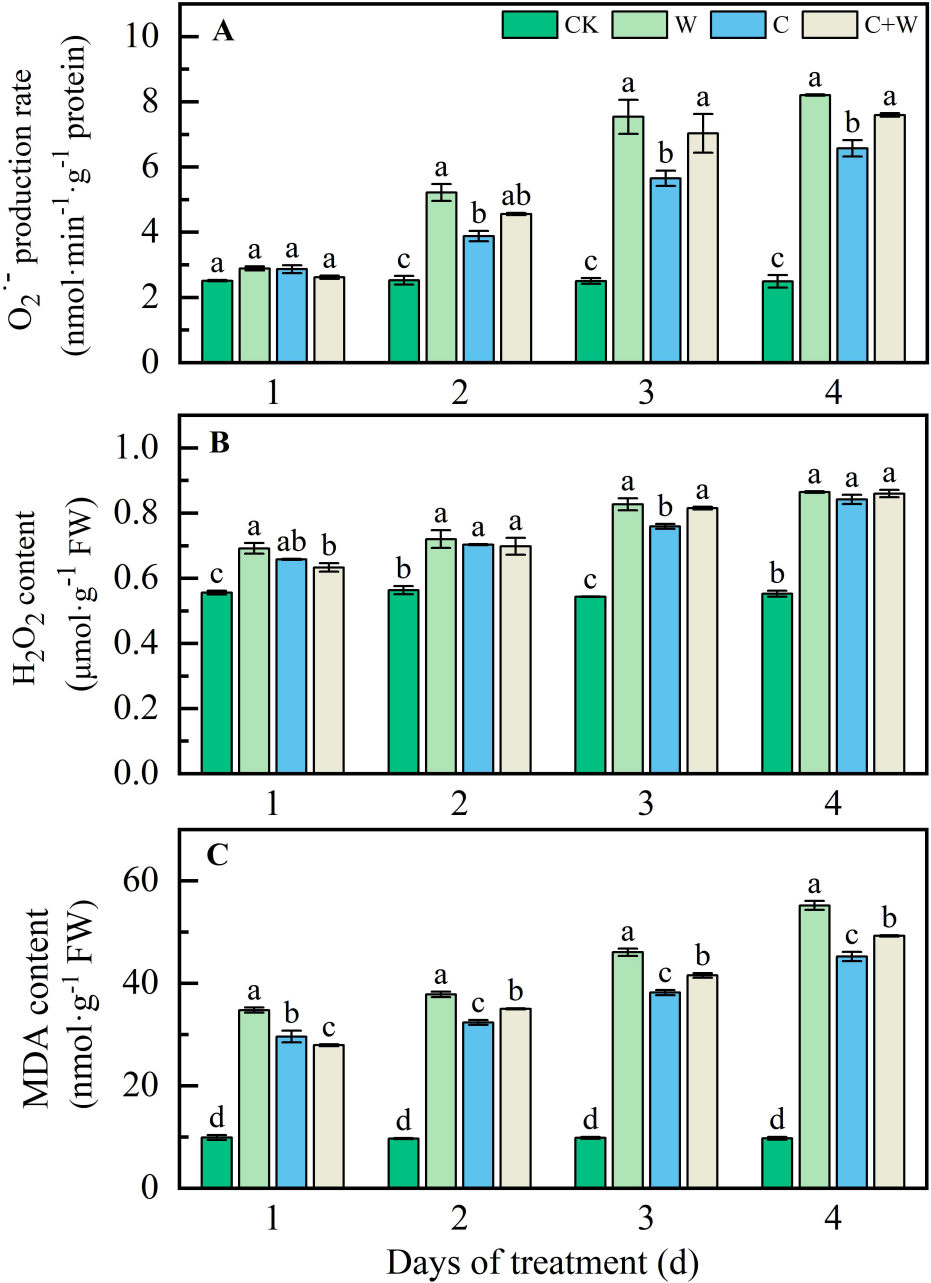
O_2_^·-^ **(A)**, H_2_O_2_ **(B)**, and MDA **(C)** level change of LXD leaves in response to single or combined stress of chilling and waterlogging at flowering stage. CK, natural conditions; W, waterlogging; C, chilling at an average temperature of 15°C; C+W, combined chilling and waterlogging. Data are represented as mean ± SD of three replicates, lowercase letters represent significant differences between the treatment and control according to LSD (*p*<0.05).

The single or combined stress of chilling and waterlogging caused severe oxidative damage to LXD leaves, exhibited by high levels of reactive oxygen species (O_2_^·-^, H_2_O_2_) and MDA with prolonged stress duration (**Figure 1**). The O_2_^·-^ production rate, H_2_O_2_ and MDA content followed the same pattern from 2 d to the end of treatment, W>C+W>C>CK. At 4 d after treatment, compared with CK, the O_2_^·-^ production rate of W, C and C+W treatments in LXD leaves was significantly increased by 2.30, 1.64 and 2.05 times, respectively (**Figure 1A**); the H_2_O_2_ content was significantly increased by 56.37%, 52.20%, and 55.46%, respectively (**Figure 1B**); the MDA content was significantly increased by 4.67, 3.65, 4.06 times, respectively (**Figure 1C** **).**

#### 3.1.2 Photosynthetic characters response

**Figure 2:**
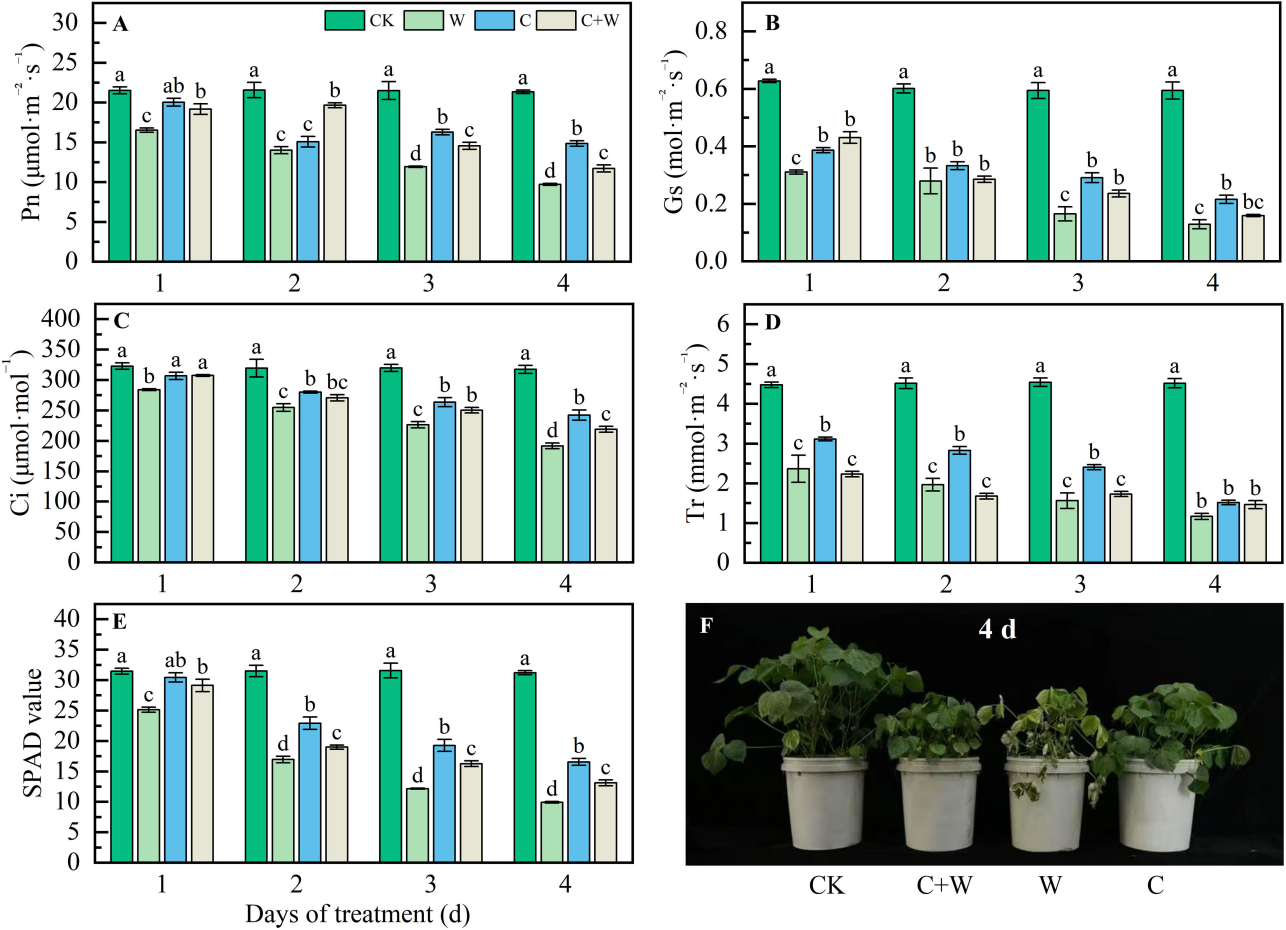
Photosynthetic characters response of LXD leaves to single or combined stress of chilling and waterlogging at flowering stage. **(A)** Pn; **(B)** Gs; **(C)** Ci; **(D)** Tr; **(E)** SPAD value; **(F)** phenotype of plants on the 4 d after stress. CK, natural conditions; W, waterlogging; C, chilling at an average temperature of 15°C; C+W, combined chilling and waterlogging. Data are represented as mean ± SD of three replicates, lowercase letters represent significant differences between the treatment and control according to LSD (*p*<0.05).

As shown in **Figure 2**, chilling and waterlogging stress significantly inhibited photosynthesis; compared with CK, the Pn, Gs, Ci, Tr, and SPAD values of LXD leaves were significantly reduced with the extension of stress time. waterlogging stress showed the most significant inhibitory effect. For example, at the end of the trial, the SPAD value of W treatment in LXD leaves decreased by 68.27% compared with CK (**Figures 2E, F**). While combined treatment of chilling and waterlogging showed an alleviation effect, C+W treatment relieved 20.58%, 23.51%, 14.10%, 25.30%, and 32.40% reduction in Pn, Gs, Ci, Tr, and SPAD values compared with W treatment, respectively, although still lower than those under chilling treatment.

### 3.2 Phytohormones of LXD leaves in modulating chilling and waterlogging stress

**Figure 3:**
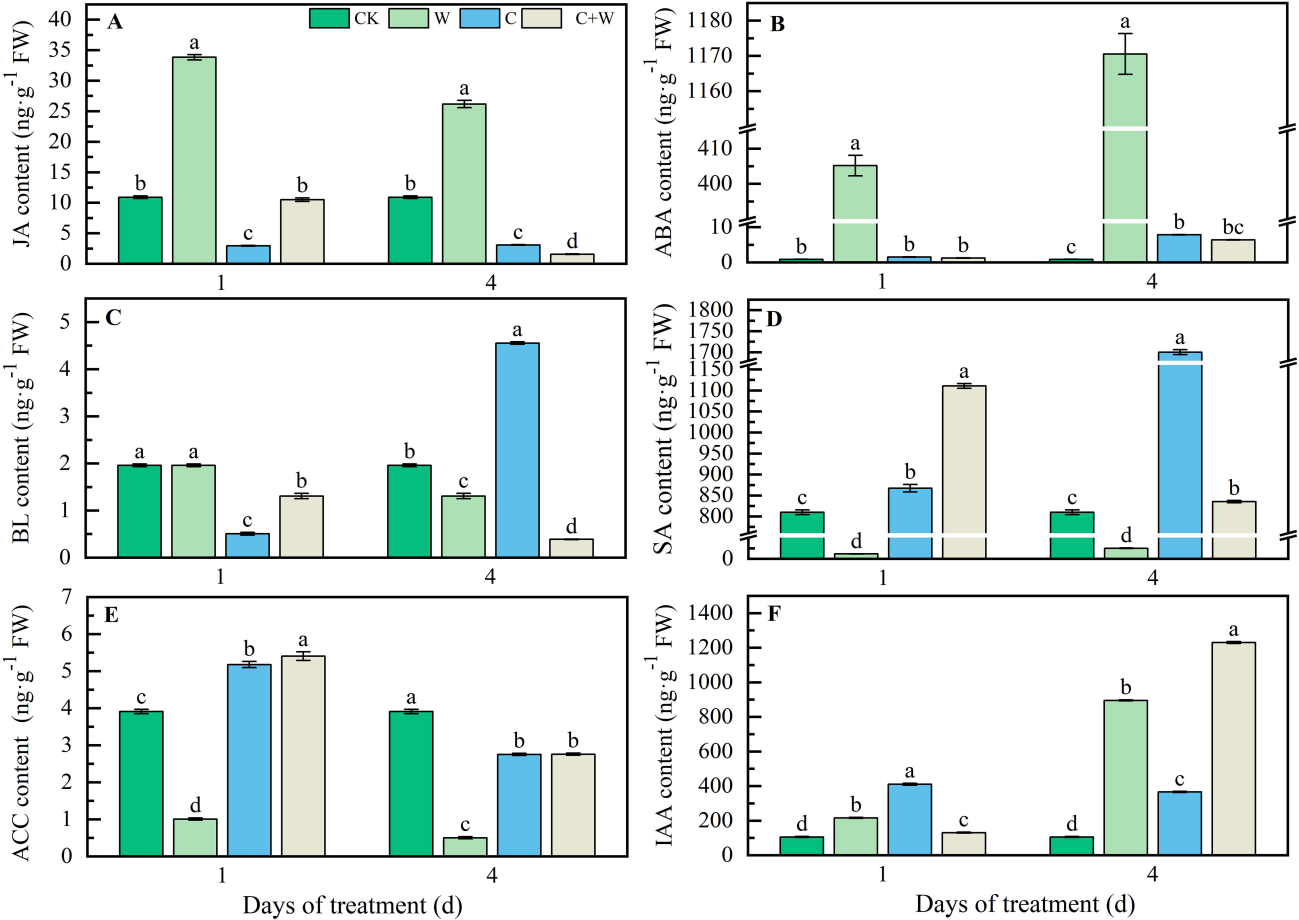
Phytohormones response of LXD leaves to single or combined stress of chilling and waterlogging at flowering stage. **(A)** JA content; **(B)** ABA content; **(C)** BL content; **(D)** SA content; **(E)** ACC content; **(F)** IAA content. CK, natural conditions; W, waterlogging; C, chilling at an average temperature of 15°C; C+W, combined chilling and waterlogging. Data are represented as mean ± SD of three replicates, lowercase letters represent significant differences between the treatment and control according to LSD (*p*<0.05).

The change in phytohormone accumulation of LXD leaves under different stresses are shown in **Figure 3**. waterlogging stress significantly promoted the increase of Jasmonic acid (JA) content. At 1 d and 4 d after stress, the JA content in the W treatment was increased by 2.11 times and 1.40 times, respectively, compared with CK. However, the JA content of C and C+W treatments was lower than that of CK (**Figure 3A**). Both single and compound stress significantly increased Abscisic acid (ABA) content, especially the surprising accumulation of ABA in LXD leaves after waterlogging treatment. At the 4 d after stress, the ABA content of W, C, and C+W treatments increased by 1385.42 times, 8.26 times, and 6.56 times, compared with CK, respectively (**Figure 3B**). The Brassinolide (BL) content in the C treatment was 132.44% higher than that in CK (**Figure 3C**). C and C+W treatments accumulated Salicylic acid (SA) content, while W treatment consistently had lower SA and 1-aminocyclopropane-1-carboxylic acid (ACC) content than CK at 1 d and 4 d after treatment (**Figures 3D, E**). Both single and combined chilling and waterlogging stresses significantly accelerated Indole-3-acetic acid (IAA) content accumulation, particularly the IAA content of W, C, and C+W treatment showed 751.76%, 248.18%, and 1070.64% increase compared with CK at 4 d after treatment, respectively (**Figure 3F**).

### 3.3 Metabolomics analysis of LXD leaves under chilling and waterlogging stress

#### 3.3.1 Principal component analysis

**Figure 4:**
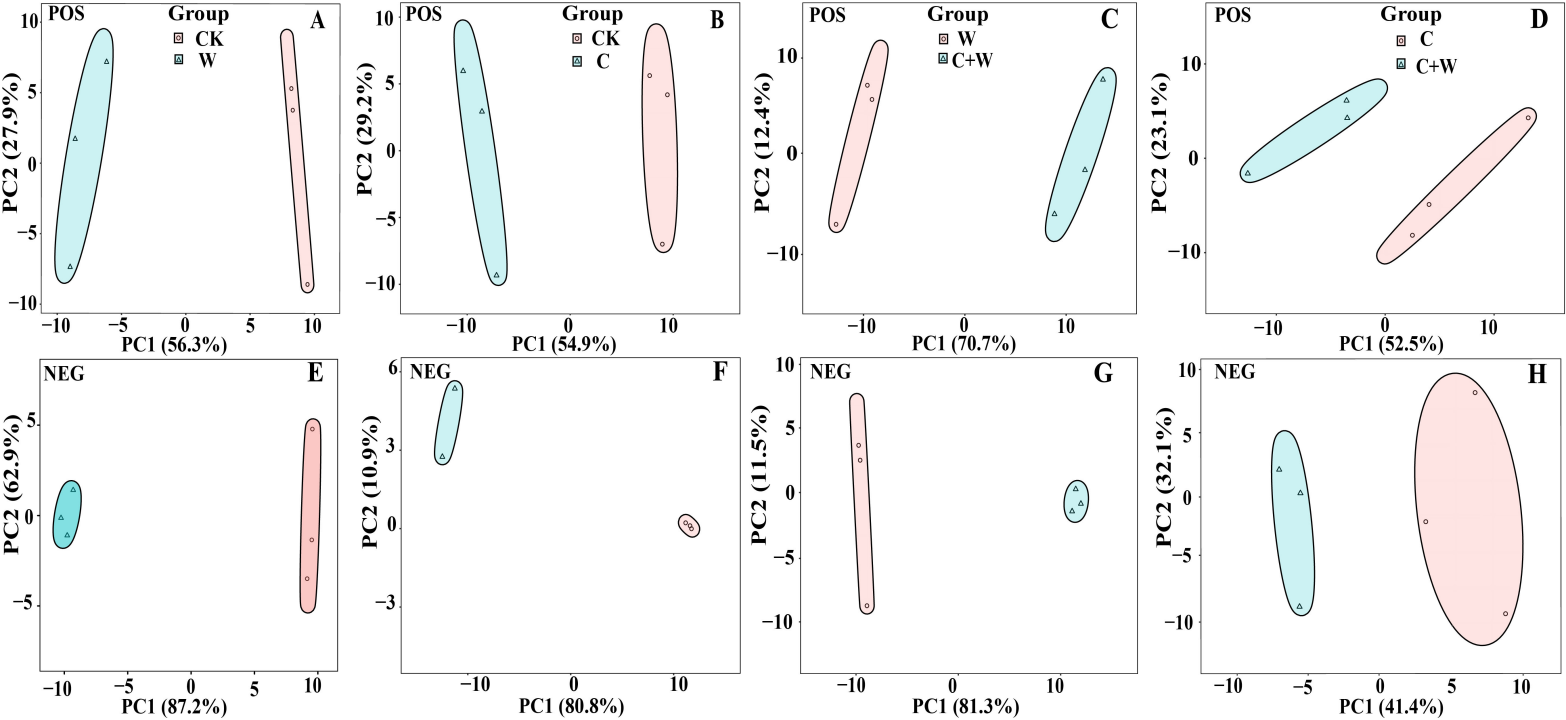
PCA model score chart in the comparison groups of W vs. CK in the positive ion modes **(A)**; C vs. CK in the positive ion modes **(B)**; C+W vs. W in the positive ion modes **(C)**; C+W vs. C in the positive ion modes **(D)**; W vs. CK in the negative ion modes **(E)**; C vs. CK in the negative ion modes **(F)**; C+W vs. W in the negative ion modes **(G)**; C+W vs. C in the negative ion modes **(H)**. CK, natural conditions; W, waterlogging; C, chilling at an average temperature of 15°C; C+W, combined chilling and waterlogging.

To investigate the metabolite profiles of LXD leaves under single or combined stress of chilling and waterlogging, four leaf samples with three replicates each were divided into four groups (W vs. CK, C vs. CK, C+W vs. W, C+W vs. C). These groups were analyzed by non-targeted LC-MS (non-targeted liquid chromatography-mass spectrometry) to determine metabolomic changes in LXD leaves. According to PCA, all samples were within the 95% confidence interval, and CK, W, C and C+W groups had clear boundaries. PCA revealed significant variation in metabolites between W and CK (**Figures 4A, E**), C and CK (**Figures 4B, F**), C+W and W (**Figures 4C, G**), as well as between C+W and C (**Figures 4D, H**) in both positive or negative ion mode. These results indicated significant differences in overall metabolites among different groups, and the data were reliable and reproducible, which could be used for subsequent analysis.

#### 3.3.2 The screening of differentially expressed metabolites

OPLS-DA was performed on the identified metabolites to clarify the differentially expressed metabolites (DEM) of single or combined stress of chilling and waterlogging in LXD leaves. The results showed significant differences between W and CK; similarly, C and CK were entirely separated, as well as C+W and W, and C+W and C (**Supplementary Figure S2**). In both positive and negative ion modes, cross-validation (n=200) results indicated that the Q2 values of the four comparison groups were all greater than 0.5, indicating that the models were reliable and without evidence of overfitting (**Supplementary Figure S3**).

**Figure 5:**
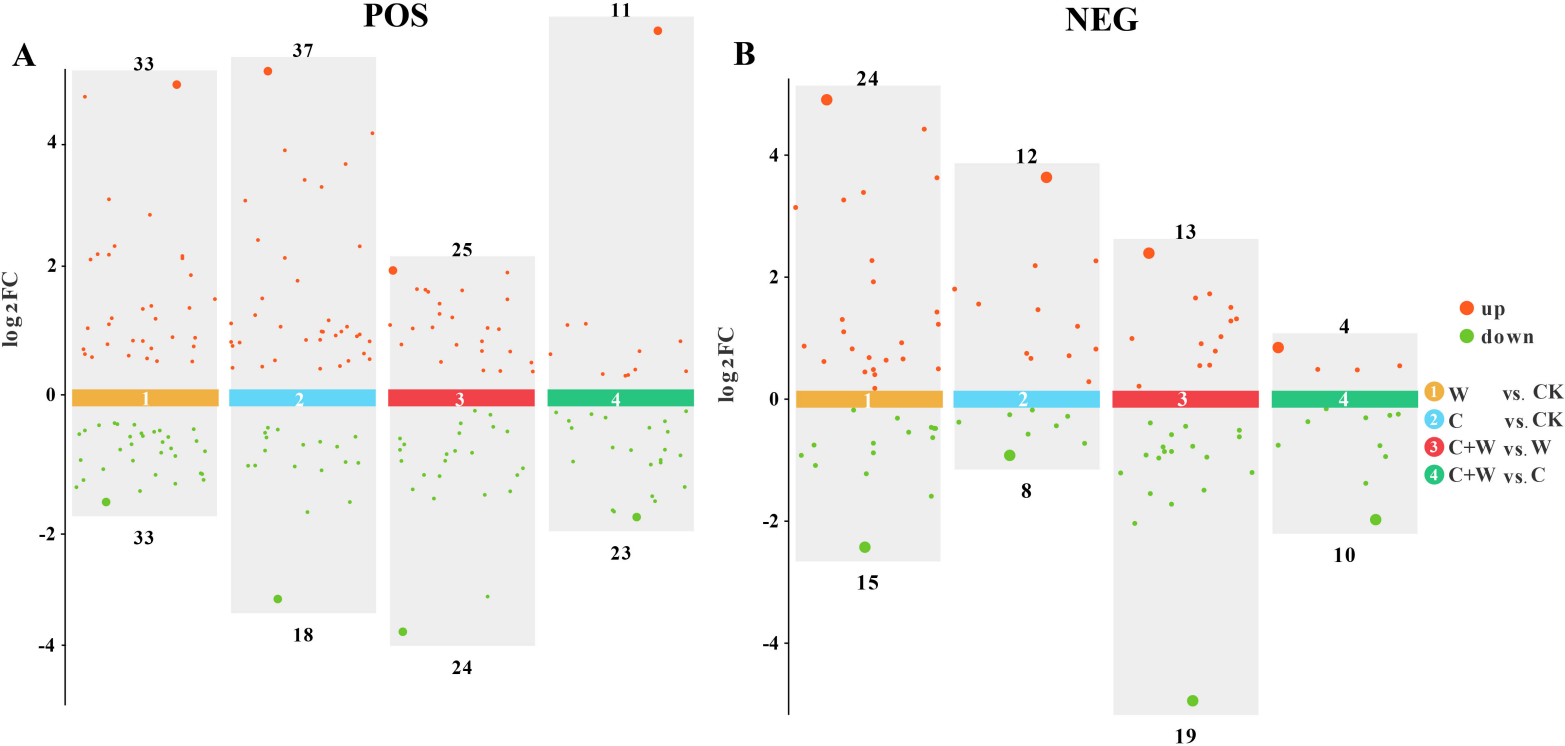
Scatter diagram of DEMs in the comparison groups of W vs. CK, C vs. CK, C+W vs. W and C+W vs. C in the positive **(A)** and negative **(B)** ion modes. CK, natural conditions; W, waterlogging; C, chilling at an average temperature of 15°C; C+W, combined chilling and waterlogging.

The DEMs in W vs. CK, C vs. CK, C+W vs. W and C+W vs. C groups were screened based on the OPLS-DA model, using the VIP (variable importance in projection) ≥1 and *p*<0.05 as screening criteria. In addition, the DEMs of each group were analyzed in the positive or negative ion mode. Results showed that a total of 66 DEMs were identified in the positive ion mode of W vs. CK, including 29 up-regulated DEMs and 30 down-regulated DEMs, and 39 DEMs were identified in the negative ion mode, including 24 up-regulated DEMs and 15 down-regulated DEMs. In the C vs. CK group, in the positive ion mode, a total of 55 DEMs were detected, with 37 DEMs up-regulated and 18 DEMs down-regulated; in the negative ion mode, 20 DEMs were detected, with 12 DEMs up-regulated and 8 DEMs down-regulated. In the comparison groups of C+W vs. W, a total of 59 and 32 DEMs were detected in the positive and negative ion mode, respectively, with 25 and 13 DEMs up-regulated, 24 and 19 DEMs down-regulated, respectively. In the C+W vs. C group, 34 DEMs (up-regulated: 11 DEMs, down-regulated: 23 DEMs) and 14 DEMs (up-regulated: 4 DEMs, down-regulated: 10 DEMs) were identified in the positive and negative ion mode, respectively (**Figure 5**). In the comparison groups, the DEMs mainly contained Amino acids, peptides, and analogues (8), Carbohydrates and carbohydrate conjugates (6), Diterpenoids (11), Flavonoid glycosides (8), Tetraterpenoids (12), Lipids and lipid-like molecules (50), Organic acids and derivatives (13), Organic oxygen compounds (7), Phenylpropanoids and polyketides (15), and Organoheterocyclic compounds (9), while a large proportion of DEMs were unclassified (**Supplementary Figure S4**).

#### 3.3.3 The metabolic pathway analysis of DEMs

**Figure 6:**
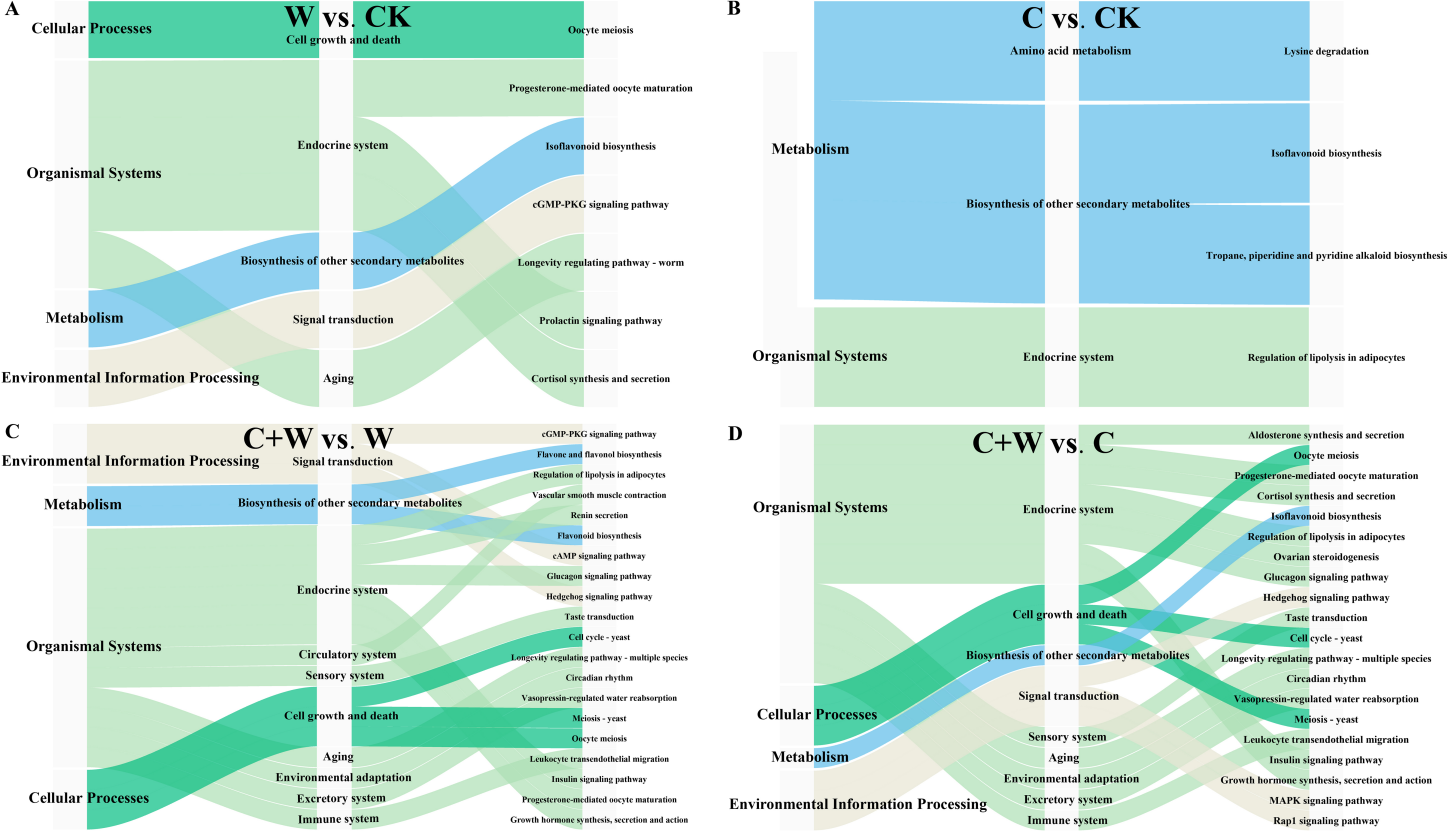
KEGG annotation analysis of DEMs in the comparison groups of W vs. CK **(A)**, C vs. CK **(B)**, C+W vs. W **(C)** and C+W vs. C **(D)**. CK, natural conditions; W, waterlogging; C, chilling at an average temperature of 15°C; C+W, combined chilling and waterlogging.

**Figure 7:**
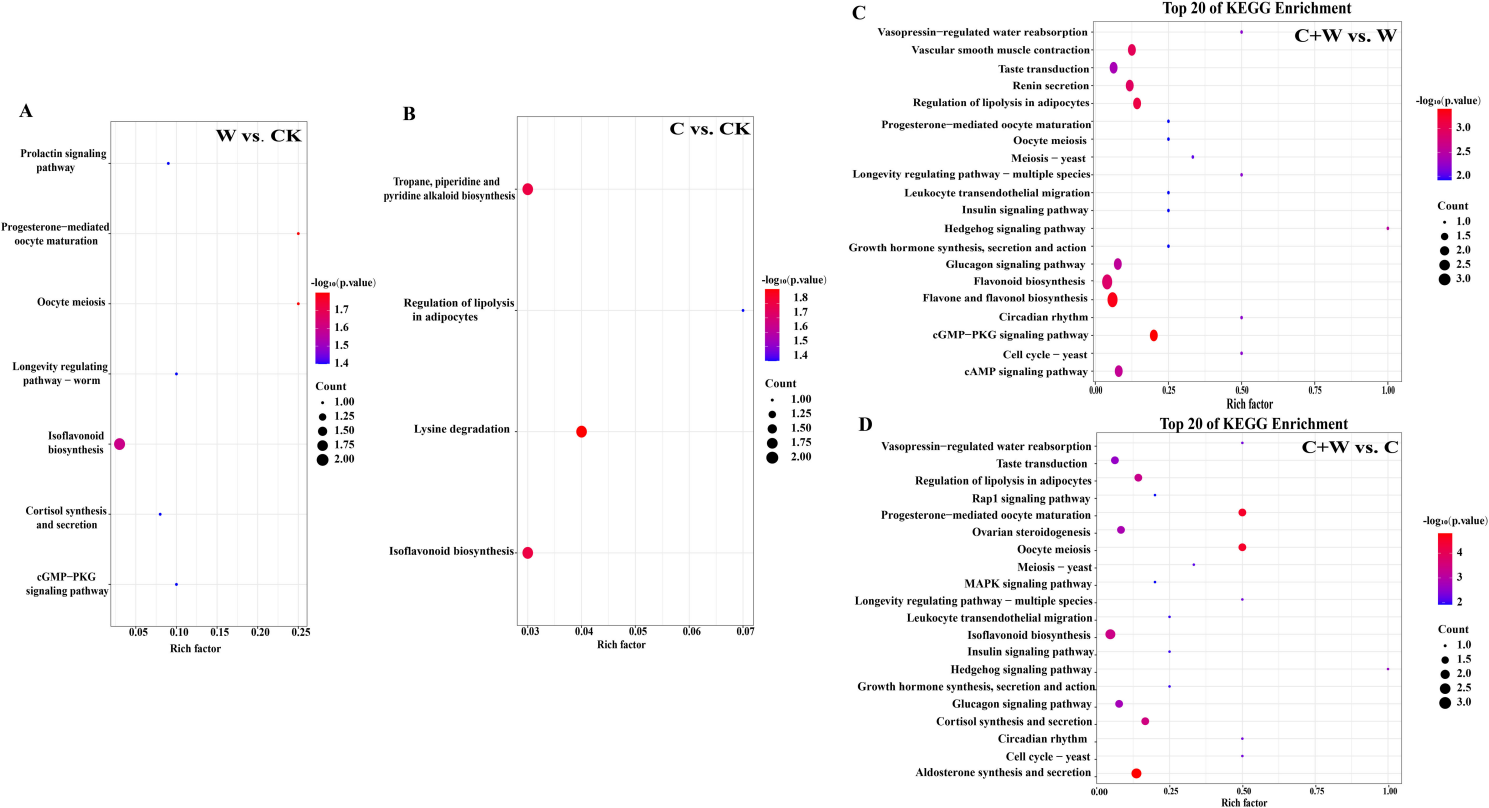
KEGG enrichment analysis of DEMs in the comparison groups of W vs. CK **(A)**, C vs. CK **(B)**, C+W vs. W **(C)** and C+W vs. C **(D)**. CK, natural conditions; W, waterlogging; C, chilling at an average temperature of 15°C; C+W, combined chilling and waterlogging.

To clarify the functional properties of these metabolites, KEGG (Kyoto Encyclopedia of Genes and Genomes) functional annotation was performed for the DEMs in the comparison groups of W vs. CK, C vs. CK, C+W vs. W, and C+W vs. C (**Figure 6**). We found that most differential metabolites were classified into metabolic pathways related to endocrine functions (W vs. CK: 3, C vs. CK: 1, C+W vs. W: 7, C+W vs. C: 8), biosynthesis of other secondary metabolites (W vs. CK: 1, C vs. CK: 2, C+W vs. W: 1, C+W vs. C: 1), Cell Growth and Death (W vs. CK: 1, C+W vs. W: 3, C+W vs. C: 3), and Signal Transduction (W vs. CK: 1, C+W vs. W: 3, C+W vs. C: 3). Furthermore, the DEMs in the comparison groups of C+W vs. W and C+W vs. C were more diverse. KEGG enrichment analysis was performed for the DEMs of the four pairwise comparison groups (W vs. CK, C vs. CK, C+W vs. W, and C+W vs. C; (**Supplementary Table S1**). The results indicated that 7 differential metabolic pathways were significantly enriched in the W vs. CK group, with the most significant pathways related to isoflavonoid biosynthesis pathways. Apigenin (C01477, up-regulated) and Biochanin A 7-O-beta-D-glucoside-6’’-O-malonate (C12625, down-regulated) were involved in these KEGG pathways (**Figure 7A** **;** **Supplementary Figure S5**).

A total of 4 significantly enriched KEGG pathways were found in the C vs. CK group, with the most significant being lysine degradation. N6, N6, N6-Trimethyl-L-lysine (C03793, up-regulated), and pipecolic acid (C00408, up-regulated) were involved in these KEGG pathways (**Figure 7B**; **Supplementary Figure S4**). In the comparison group of C+W vs. W, the DEMs were annotated to 59 differential metabolic pathways, with the DEMs mainly enriched in flavone and flavonol biosynthesis and cGMP-PKG signaling pathways. Apigenin (C01477, down-regulated), kaempferol (C05903, down-regulated), luteolin (C01514, down-regulated), adenosine (C00212, down-regulated), and cyclic AMP (C00575, up-regulated) were involved in these pathways (**Figure 7C**; **Supplementary Figure S4**). In the comparison group of C+W vs. C, a total of 63 significantly enriched KEGG pathways were found, with the most significant being aldosterone synthesis and secretion. Corticosterone (C02140, down-regulated), Cyclic AMP (C00575, up-regulated), and Progesterone (C00410, down-regulated) were involved in these pathways (**Figure 7D** **;** **Supplementary Figure S5** **).**

### 3.4 Correlation analysis of DEMs and physiological parameters of LXD leaves

**Figure 8:**
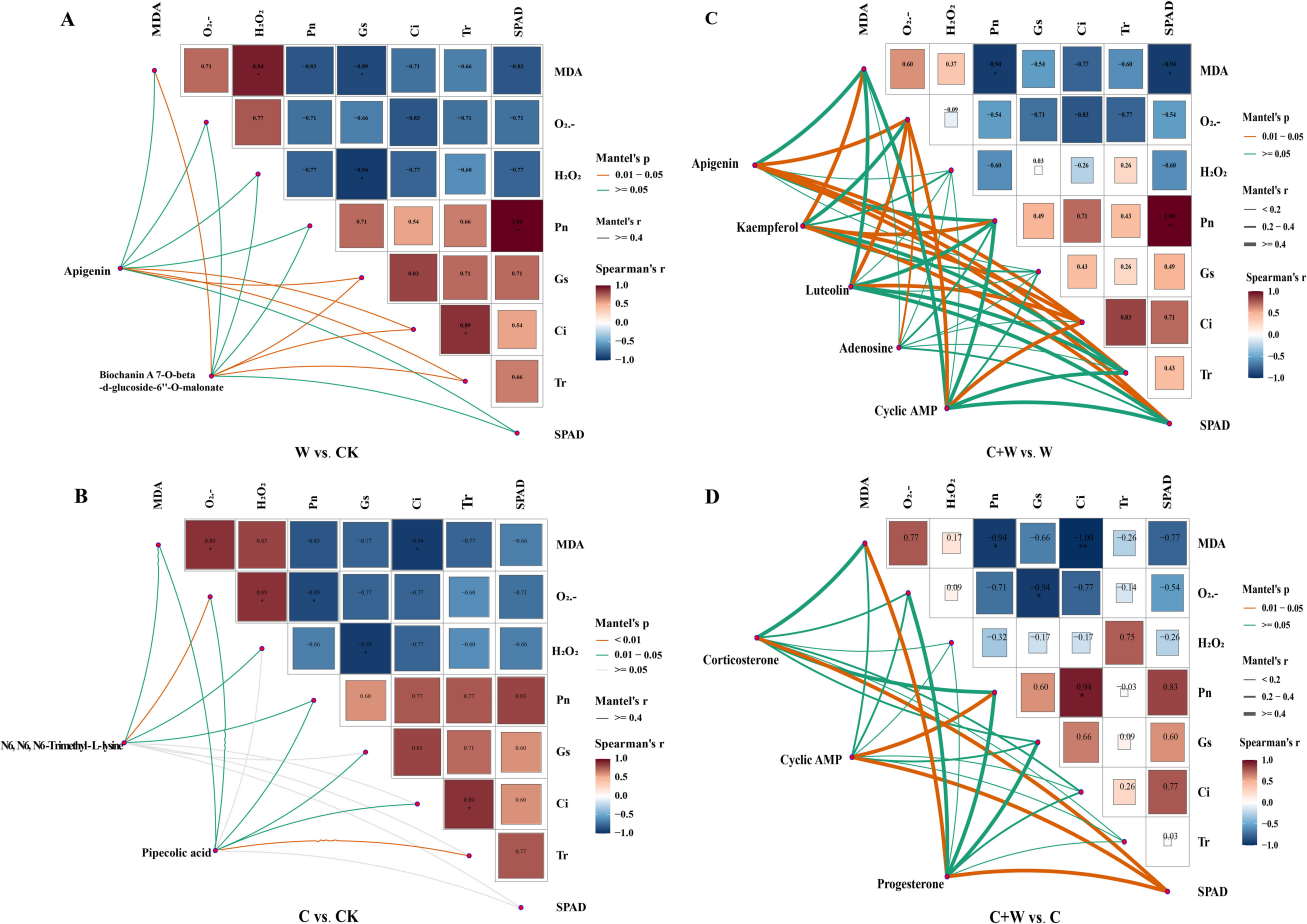
Mantel test correlation heatmap of DEMs and physiological parameters in the comparison groups of W vs. CK **(A)**, C vs. CK **(B)**, C+W vs. W **(C)** and C+W vs. C **(D)**. CK, natural conditions; W, waterlogging; C, chilling at an average temperature of 15°C; C+W, combined chilling and waterlogging.

The Mantel Test was used to analyze and visualize the correlations between DEMs in important KEGG pathways and physiological data in each comparison group. As shown in **Figure 8**, there were strong correlations between some DEMs and physiological parameters. Apigenin and Biochanin A 7-O-beta-D-glucoside-6’-O-malonate correlated positively with Gs, Ci, and Tr (*p*<0.05). Biochanin A 7-O-beta-D-glucoside-6’-O-malonate also correlated positively with MDA under the waterlogging stress condition (**Figure 8A**). Under chilling stress, N6, N6, N6-Trimethyl-L-lysine in leaves correlated positively with MDA, H_2_O_2_, and Pn (p<0.05), and correlated strongly with O_2·_⁻ (*p*<0.01). Pipecolic acid correlated positively with MDA, O_2_^·^⁻, Pn, Gs, and Ci, and correlated strongly with Tr (*p*<0.01) (**Figure 8B**). In the comparison groups of C+W vs. W, there were significant positive correlations between Apigenin and O_2_^·^⁻, Ci, Tr, and SPAD (*p*<0.05, Mantel’s r≥0.2). Kaempferol in leaves correlated strongly with MDA, Pn, Ci, and SPAD. Luteolin and Cyclic AMP correlated strongly with O_2_^·^⁻ and Ci (*p*<0.05, Mantel’s r≥0.2). Except for Apigenin, there was only a significant positive correlation between O_2_^·^⁻ and other physiological parameters (**Figure 8C**). In the C+W vs. C group, corticosterone in LXD leaves correlated strongly with SPAD (*p*<0.05, Mantel’s r≥0.2). Cyclic AMP showed strong positive correlations with Pn and SPAD values. Significant positive correlations existed between progesterone and MDA, SPAD (**Figure 8D**). In addition, we found a negative correlation between oxidative damage parameters and photosynthetic parameters by Mantel Test analysis.

### 3.5 Plant productivity changes under chilling and waterlogging stress

The yield and yield components of LXD under single or combined stress of chilling and waterlogging are shown in **Table 1**. The particle number per plant followed the order of CK>C>C+W>W from 3 d to 4 d after treatment. The yields of LXD were significantly lower than those of CK after treatments, and the yield of W, C and C+W treatment decreased gradually with the extension of stress time, C treatment showed more severe yield loss after 3 days of processing. However, after being returned to the natural growth environment after 4 days of waterlogging, the plants did not recover and then died, so we did not obtain the yield. The yield of C and C+W treatment reduced 30.12% and 40.92% after 4 days of treatment, respectively. The ANOVA results showed that different days of stress showed a highly significant effect on yield, while there was no significant effect on yield components. Different treatments had highly significant effects on yield and 100-grain weight (*p*<0.01). Two-factor analysis indicated that different days of stress and different treatments (Day×Treatment) had a highly significant influence on 100-grain weight (*p*<0.05) and no significant impact on yield and particle number per plant.

**Table 1:** Comparison of yield and yield components of adzuki bean under single or combined stress of chilling and waterlogging.

Days of treatment (d)	Treatments	Yield (g·plant^-1^)	Particle number per plant (PCS/ plant)	100-grain weight (g)
1d	CK	5.01 ± 0.30a	36.00 ± 5.09b	13.56 ± 0.16a
	W	4.41 ± 0.18c	36.00 ± 3.52c	12.41 ± 0.52b
	C	4.08 ± 0.34d	35.00 ± 5.28d	11.20 ± 0.26c
	C+W	4.54 ± 0.21b	36.60 ± 5.56a	12.50 ± 0.24b
2d	CK	5.01 ± 0.30a	36.00 ± 5.09a	13.56 ± 0.16a
	W	4.14 ± 0.32c	34.20 ± 4.59b	11.78 ± 0.27c
	C	4.40 ± 0.31b	33.40 ± 3.91c	12.49 ± 0.37b
	C+W	3.91 ± 0.24d	26.20 ± 4.32d	12.03 ± 0.17bc
3d	CK	5.01 ± 0.30a	36.00 ± 5.09a	13.56 ± 0.16a
	W	3.51 ± 0.29d	34.60 ± 2.20c	11.29 ± 0.29b
	C	4.03 ± 0.38b	34.80 ± 2.91b	11.73 ± 0.11b
	C+W	3.67 ± 0.11c	30.20 ± 6.11d	11.50 ± 0.08b
4d	CK	5.01 ± 0.30a	36.00 ± 5.09a	13.56 ± 0.16a
	W	–	–	–
	C	3.50 ± 0.13b	29.80 ± 4.88b	11.84 ± 0.32b
	C+W	2.96 ± 0.25c	26.6 ± 5.24c	11.70 ± 0.26b
ANOVA	Day	4.23**	ns	ns
	Treatment	17.66***	ns	42.21***
	Day×Treatment	ns	ns	2.88**

The trial lasted for a total of 4 days. CK, natural conditions; W, waterlogging; C, chilling at an average temperature of 15°C; C+W, combined chilling and waterlogging. Lowercase letters represent significant differences between the treatment and control according to LSD. ns, no significance; **p<0.05; ***p<0.01.

## 4 Discussion

Photosynthesis is crucial for plant growth and productivity (De Souza et al., 2017). During this process, chlorophyll pigments capture and convert light energy to drive carbon assimilation. In the present study, both individual and combined stress conditions led to a marked reduction in photosynthetic capacity, likely attributable to stomatal limitations as well as reduced enzymatic activity in the carbon assimilation pathway (Hossain et al., 2009; Sharma et al., 2020). Specifically, significant declines in stomatal conductance and transpiration rates were observed, suggesting that stomatal closure was the primary factor limiting photosynthesis under these stress conditions (Gururani et al., 2015). Chilling stress primarily limits photosynthetic CO_2_ assimilation through stomatal closure (Huang et al., 2025). In contrast, waterlogging resulted in a more severe inhibition of photosynthesis in the present study. This inhibition was primarily attributable to rapid stomatal closure induced by ABA hyperaccumulation, together with chronic suppression of the tricarboxylic acid (TCA) cycle under anaerobic conditions. The associated hypoxia-induced energy deficits impaired ATP-dependent ion channel function, thereby exacerbating stomatal limitations (Zhang et al., 2021). In addition, the prolonged shortage of ATP broadly compromised other energy-intensive physiological processes, including photosynthesis, growth, and nutrient uptake (Malik et al., 2011; Colmer and Greenway, 2011). Interestingly, the combined waterlogging and chilling stress treatment resulted in a less severe inhibition of photosynthesis than waterlogging alone, as evidenced by higher Pn and SPAD values. This photoprotective effect may correlate with an accumulation of IAA, indicating that auxin-mediated regulation of photosystem repair and chloroplast stabilization alleviates combined stress damage (Goodger et al., 2005). Furthermore, elevated IAA levels coordinated apigenin biosynthesis with cAMP signaling activation, suggesting that adzuki bean prioritizes chloroplast maintenance over vegetative growth under concurrent waterlogging and chilling stress (De Castro et al., 2022).

Abiotic stresses could trigger reactive oxygen species, leading to oxidative stress through free radical accumulation. Generally, elevated H_2_O_2_ levels disrupt cellular membrane integrity, resulting in electrolyte leakage and impaired metabolic functions (Xu et al., 2024). In the present study, oxidative stress dynamics showed distinct stress-specific patterns. Waterlogging triggered systemic ROS propagation via hypoxia-induced mitochondrial dysfunction, initiating root ethanol fermentation and resulting in cytoplasmic acidosis and membrane damage, consistent with mechanisms observed in flood-sensitive species (Zhou et al., 2020). In contrast, chilling stress localized oxidative damage to chloroplasts, mitigated by lysine catabolism activation, which served dual roles as membrane stabilizers and NADPH regenerators (Walsh et al., 2018). MDA levels in adzuki bean leaves showed partial alleviation under combined stress. The antagonistic interaction under combined stress reflected a metabolic trade-off strategy (Liu and Jiang, 2015; Renziehausen et al., 2024), involving prioritized allocation toward apigenin-derived isoflavonoid biosynthesis versus glutathione regeneration alongside ABA-IAA hormonal crosstalk (Mahouachi et al., 2007). This metabolic adjustment likely contributed to the reduction in lipid peroxidation under conditions of reduced overall antioxidant capacity, suggesting the activation of energy-efficient ROS detoxification pathways (Rui et al., 2016).

Phytohormonal crosstalk served as a pivotal regulator of stress cross-adaptation. While waterlogging-induced ABA dominance exacerbated oxidative damage through SA suppression and impaired aerenchyma formation combined stress activated IAA-SA synergy. The observed IAA increase facilitated apoplastic H_2_O_2_ detoxification and contributed to membrane stabilization. In parallel, SA accumulation potentially primed systemic acquired resistance, a mechanism previously documented in waterlogging-tolerant legumes (Gururani et al., 2015). The observed hormone profile changes indicate adaptive crosstalk between auxin and ABA pathways, possibly reducing ABA-associated stress responses under combined stress conditions. Metabolomic profiling further uncovered stress-specific adaptation strategies. Waterlogging enriched isoflavonoid biosynthesis, consistent with their dual role in ROS scavenging and hypoxia signaling. Conversely, chilling activated lysine degradation pathways, producing compatible solutes to counteract membrane rigidification, which is distinct from the GABA accumulation typically observed in cold-tolerant cereals (Mou et al., 2021). The combined stress uniquely activated cAMP-PKG signaling (**Figure 7C**), which may coordinate stomatal adjustment with antioxidant synthesis, as evidenced by strong cAMP-SPAD correlations.

Extreme weather events severely impair crop growth through physiological stress or directly induce mortality during critical developmental stages, ultimately resulting in substantial yield losses (Liu et al., 2022). In the present study, chilling and waterlogging stresses significantly reduced adzuki bean yield, with prolonged waterlogging (4 d treatment) resulting in complete yield loss due to plant mortality after re-exposure to natural environmental conditions. This catastrophic yield reduction under waterlogging highlights the urgent need to develop climate-resilient adzuki bean cultivars. Several potential mitigation strategies are indicated by the current findings. Engineering apigenin biosynthesis to enhance antioxidant capacity, application of methyl jasmonate to improve hypoxia tolerance, and optimization of root microbiome communities to support lysine metabolism represent promising approaches. The observed strong correlation between leaf cyclic cAMP content and chlorophyll levels further supports the feasibility of these strategies. Despite these insights, critical knowledge gaps remain. In particular, the role of cAMP–PKG signaling in regulating auxin transporters requires further investigation. Moreover, the epigenetic inheritance mechanisms underlying stress memory transmission across generations are still poorly understood. Future research should employ multi-generational omics-based approaches to elucidate the molecular basis of these stress adaptation mechanisms.

## 5 Conclusion

This study elucidated the intricate physiological and metabolic adaptation mechanisms of the adzuki bean to combined chilling-waterlogging stress during flowering. The findings demonstrate that the adzuki bean exhibits antagonistic effects on photosynthesis and oxidative damage under the combined stress. The combined stress reducd elevated ROS and MDA levels, while simultaneously enriching the cGMP-PKG signaling pathway, flavone/flavonol biosynthesis pathways and secretion. These enriched pathways enhance antioxidant defenses and improve photosynthetic efficiency. Additionally, the combined stress significantly decreases ABA accumulation while increasing IAA levels. Together, these regulatory pathways alleviate the inhibitory effects on photosynthesis and cellular damage, ultimately reducing yield loss.

## Data Availability

The original contributions presented in the study are included in the article/**Supplementary Material**. Further inquiries can be directed to the corresponding authors.

## Supplementary material

Supplementary Figure S1 Changes in temperature during one day.

Supplementary Figure S2 OPLS-DA model score map.

Supplementary Figure S3 Response sequencing verification diagram of the OPLS-DA model.

Supplementary Figure S4 Bar plot of the differential metabolites from each comparison group.

Supplementary Figure S5 Heatmap of the DEMs in the most signifcant KEGG pathways.

Supplementary Table S1 Total KEGG pathway of DEMs in the comparison groups.

## References

[B1] Colmer T. D. Greenway H. (2011). Ion transport in seminal and adventitious roots of cereals during O2 deficiency. J. Exp. Bot. 62, 39–57. doi: 10.1093/jxb/erq271 20847100

[B2] De Castro J. Hill R. D. Stasolla C. Badea A. (2022). Waterlogging stress physiology in barley. Agronomy 12, 780. doi: 10.3390/agronomy12040780

[B3] De Souza A. P. Massenburg L. N. Jaiswal D. Cheng S. Shekar R. Long S. P. (2017). Rooting for cassava: insights into photosynthesis and associated physiology as aroute to improve yield potential. New Phytol. 213, 50–65. doi: 10.1111/nph.14250 27778353

[B4] Ding Y. Shi Y. Yang S. (2019). Advances and challenges in uncovering cold tolerance regulatory mechanisms in plants. New Phytol. 222, 1690–1704. doi: 10.1111/nph.15696 30664232

[B5] Fukao T. Barrera-Figueroa B. E. Juntawong P. Peña-Castro J. M. (2019). Submergence and waterlogging stress in plants: a review highlighting research opportunities and understudied aspects. Front. Plant Sci. 10. doi: 10.3389/fpls.2019.00340 PMC6439527 30967888

[B6] Goodger J. Q. D. Sharp R. E. Marsh E. L. Schachtman D. P. (2005). Relationships between xylem sap constituents and leaf conductance of well-watered and water-stressed maize across three xylem sap sampling techniques. J. Exp. Bot. 56, 2389–2400. doi: 10.1093/jxb/eri231 16043455

[B7] Guo Z. Cai L. Liu C. Chen Z. Guan S. Ma W.. (2022). Low-temperature stress affects reactive oxygen species, osmotic adjustment substances, and antioxidants in rice (Oryza sativa L.) at the reproductive stage. Sci. Rep. 12, 6224. doi: 10.1038/s41598-022-10420-8 35418703 PMC9008029

[B8] Gururani M. A. Venkatesh J. Tran L. S. P. (2015). Regulation of photosynthesis during abiotic stress-induced photoinhibition. Mol. Plant (Cell Press) 8, 1304–1320. doi: 10.1016/j.molp.2015.05.005 25997389

[B9] Hasegawa T. Sakurai G. Fujimori S. Takahashi K. Hijioka Y. Masui T. (2021). Extreme climate events increase risk of global food insecurity and adaptation needs. Nat. Food 8, 2. doi: 10.1038/s43016-021-00335-4 37118168

[B10] Heilemann J. Klassert C. Samaniego L. Thober S. Marx A. Boeing F.. (2024). Projecting impacts of extreme weather events on crop yields using LASSO regression. Weather Climate Extremes 46, 100738. doi: 10.1016/j.wace.2024.100738

[B11] Hossain Z. López-Climent M. F. Arbona V. Pérez-Clemente R. M. Gómez-Cadenas A. (2009). Modulation of the antioxidant system in citrus under waterlogging and subsequent drainage. J. Plant Physiol. 166, 1391–1404. doi: 10.1016/j.jplph.2009.02.012 19362387

[B12] Hu H. Feng N. Shen X. Zhao L. Zheng D. (2022). Transcriptomic analysis of vigna radiata in response to chilling stress and uniconazole application. BMC Genomics 23, 1–14. doi: 10.1186/s12864-022-08443-6 35287570 PMC8922894

[B13] Huang S. Wang H. Liu S. Lu S. Hua J. Zou B. (2025). Ethylene antagonizes ABA and inhibits stomatal closure and chilling tolerance in rice. J. Exp. Bot. eraf052. doi: 10.1093/jxb/eraf052 39912223

[B14] Li L.-c. He Q.-s. Harrison M. T. Shi Y. Feng P.-y. Wang B.. (2025). Knowledge-guided machine learning for improving crop yield projections of waterlogging effects under climate change. Resources Environ. Sustainability 19, 100185. doi: 10.1016/j.resenv.2024.100185

[B15] Liu K. Harrison M. T. Archontoulis S. V. Huth N. Yang R. Liu D. L.. (2021). Climate change shifts forward flowering and reduces crop waterlogging stress. Environ. Res. Lett. 16, 094017. doi: 10.1088/1748-9326/ac1b5a

[B16] Liu K. Harrison M. T. Shabala S. Meinke H. Ahmed I. Zhang Y.-b.. (2020). The state of the art in modeling waterlogging impacts on plants: what do we know and what do we need to know. Earth’s Future 8, e2020EF001801. doi: 10.1029/2020EF001801

[B17] Liu K. Harrison M. T. Wang B. Yang R. Yan H.-l. Zou J.. (2022). Designing high-yielding wheat crops under late sowing: a case study in southern China. Agron. Sustain. Dev. 42, 29. doi: 10.1007/s13593-022-00764-w

[B18] Liu K. Harrison M. T. Yan H.-l. Liu D. L. Meinke H. Hoogenboom G.. (2023). Silver lining to a climate crisis in multiple prospects for alleviating crop waterlogging under future climates. Nat. Commun. 14, 765. doi: 10.1038/s41467-023-36129-4 36765112 PMC9918449

[B19] Liu M. Jiang Y. (2015). Genotypic variation in growth and metabolic responses of perennial ryegrass exposed to short-term waterlogging and submergence stress. Plant Physiol. Biochem. 95, 57–64. doi: 10.1016/j.plaphy.2015.07.008 26188499

[B20] Mahouachi J. Arbona V. Gómez-Cadenas A. (2007). Hormonal changes in papaya seedlings subjected to progressive water stress and re-watering. Plant Growth Regul. 53, 43–51. doi: 10.1007/s10725-007-9202-2

[B21] Malik A. Islam A. Colmer T. (2011). Transfer of the barrier to radial oxygen loss in roots of Hordeum marinum to wheat (Triticum aestivum): evaluation of four H. marinum–wheat amphiploids. New Phytol. 190, 499–508. doi: 10.1111/j.1469-8137.2010.03519.x 21054414

[B22] Manghwar H. Hussain A. Alam I. Khoso M. A. Ali Q. Liu F. (2024). Waterlogging stress in plants: unraveling the mechanisms and impacts on growth, development, and productivity. Environ. Exp. Bot. 224, 105824. doi: 10.1016/j.envexpbot.2024.105824

[B23] Mou D. Yang F. Kazhuo C.-r. Qi X.-z. Gu Z.-h. Xie J.-x.. (2021). Metabonomic analysis of caucasian clover in response to low-temperature stress of different cooling modes. Acta Agrestia Sin. 29, 1877–1884. doi: 10.11733/j.issn.1007-0435.2021.09.002

[B24] Ohkawa H. Ohishi N. Yagi K. (1979). Assay for lipid peroxides in animal tissues by thiobarbituric acid reaction. Analytical Biochem. 95, 351–358. doi: 10.1016/0003-2697(79)90738-3 36810

[B25] Park J. Jung J. H. (2024). Revalidation of the ICE1–CBF regulatory model in arabidopsis cold stress response. J. Plant Biol. 67, 391–398. doi: 10.1007/s12374-024-09440-w

[B26] Renziehausen T. Frings S. Schmidt-Schippers R. (2024). ‘Against all floods’: plant adaptation to flooding stress and combined abiotic stresses. Plant J. 117 (6), 1836–1855. doi: 10.1111/tpj.16614 38217848

[B27] Rezaei E. E. Webber H. Asseng S. Boote K. J. Durand J.-L. Ewert F.. (2023). Climate change impacts on crop yields. Nat. Rev. Earth Environ. 4, 831–846. doi: 10.1038/s43017-023-00491-0

[B28] Rui G. Ji Z. Fan Y. Feng L. I. Hao-Ru L. I. Xu X.. (2016). Growth metabolism of wheat under drought stress at the jointing-booting stage. Chin. J. Plant Ecol. 40, 1319–1327. doi: 10.17521/cjpe.2016.0107

[B29] Sharma A. Kumar V. Shahzad B. Ramakrishnan M. Singh Sidhu G. P. Bali A. S.. (2020). Photosynthetic response of plants under different abiotic stresses: a review. J. Plant Growth Regul. 39, 509–531. doi: 10.1007/s00344-019-10018-x

[B30] Thapa R. Tabien R. E. Johnson C. D. Septiningsih E. M. (2023). Comparative transcriptomic analysis of germinating rice seedlings to individual and combined anaerobic and cold stress. BMC Genomics 24, 185. doi: 10.1186/s12864-023-09262-z 37024819 PMC10080786

[B31] Walsh C. T. Tu B. P. Tang Y. (2018). Eight kinetically stable but thermodynamically activated molecules that power cell metabolism. Chem. Rev. 118, 1460–1494. doi: 10.1021/acs.chemrev.7b00510 29272116 PMC5831524

[B32] Xiang H. Liang X. Wang S. Wang X. He N. Dong X.. (2024). Foliar spraying exogenous ABA resists chilling stress on adzuki beans (*Vigna angularis*). PloS One 19, e0304628. doi: 10.1371/journal.pone.0304628 39250484 PMC11383210

[B33] Xu Z.-y. Ye L.-z. Shen Q.-f. Zhang G.-p. (2024). Advances in the study of waterlogging tolerance in plants. J. Integr. Agric. 23, 2877–2897. doi: 10.1016/j.jia.2023.12.028

[B34] Yang R. Wang C.-h. Yang Y.-m. Harrison M. T. Zhou M.-x. Liu K. (2024). Implications of soil waterlogging for crop quality: a meta-analysis. Eur. J. Agron. 161, 127395. doi: 10.1016/j.eja.2024.127395

[B35] Zhang Y. Liu G. Dong H. Li C. (2021). Waterlogging stress in cotton: damage, adaptability, alleviation strategies, and mechanisms. Crop J. 9, 257–270. doi: 10.1016/j.cj.2020.08.005

[B36] Zhou W. Chen F. Meng Y. Chandrasekaran U. Shu K. (2020). Plant waterlogging/flooding stress responses: from seed germination to maturation. Plant Physiol. Biochem. 148, 228–236. doi: 10.1016/j.plaphy.2020.01.020 31981875

