# Supplementary figures and images for: Physiological and metabolomic responses of adzuki bean (Vigna angularis) to individual and combined chilling and waterlogging stress
Source: Front Plant Sci. 2025 May 22;16:1598648. doi: 10.3389/fpls.2025.1598648 (PMC12138264; doi:10.3389/fpls.2025.1598648)

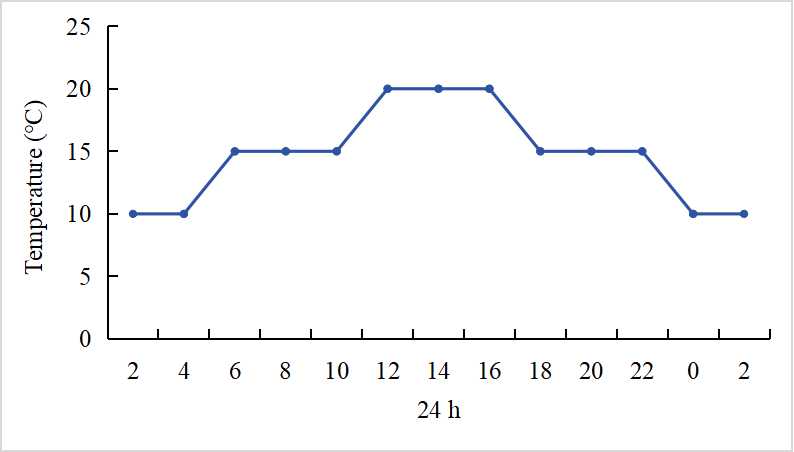

Supplement: Supplementary Figure S1 — Changes in temperature during one day. [file Image1.jpeg]

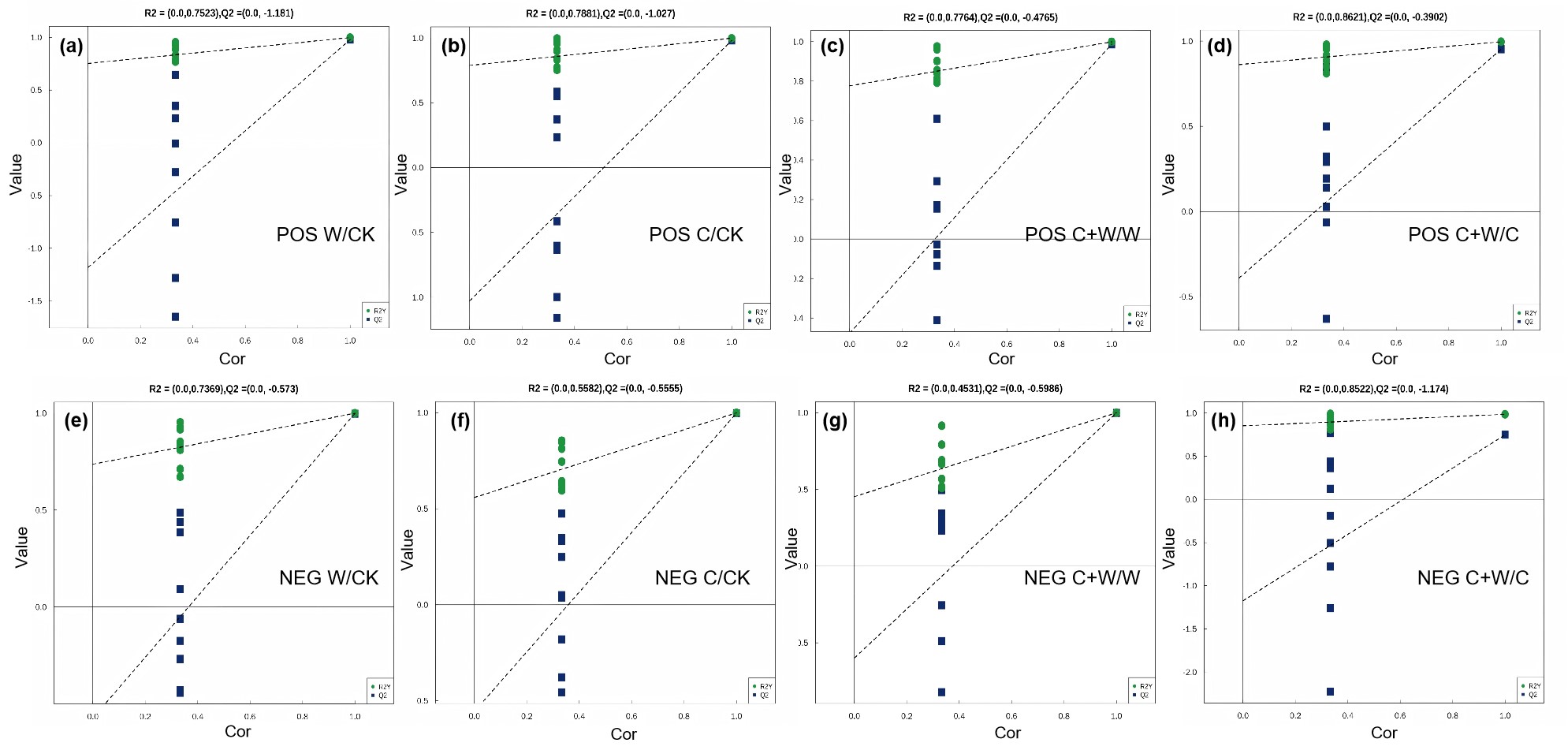

Supplement: Supplementary Figure S3 — Response sequencing verification diagram of the OPLS-DA model. [file Image3.jpeg]

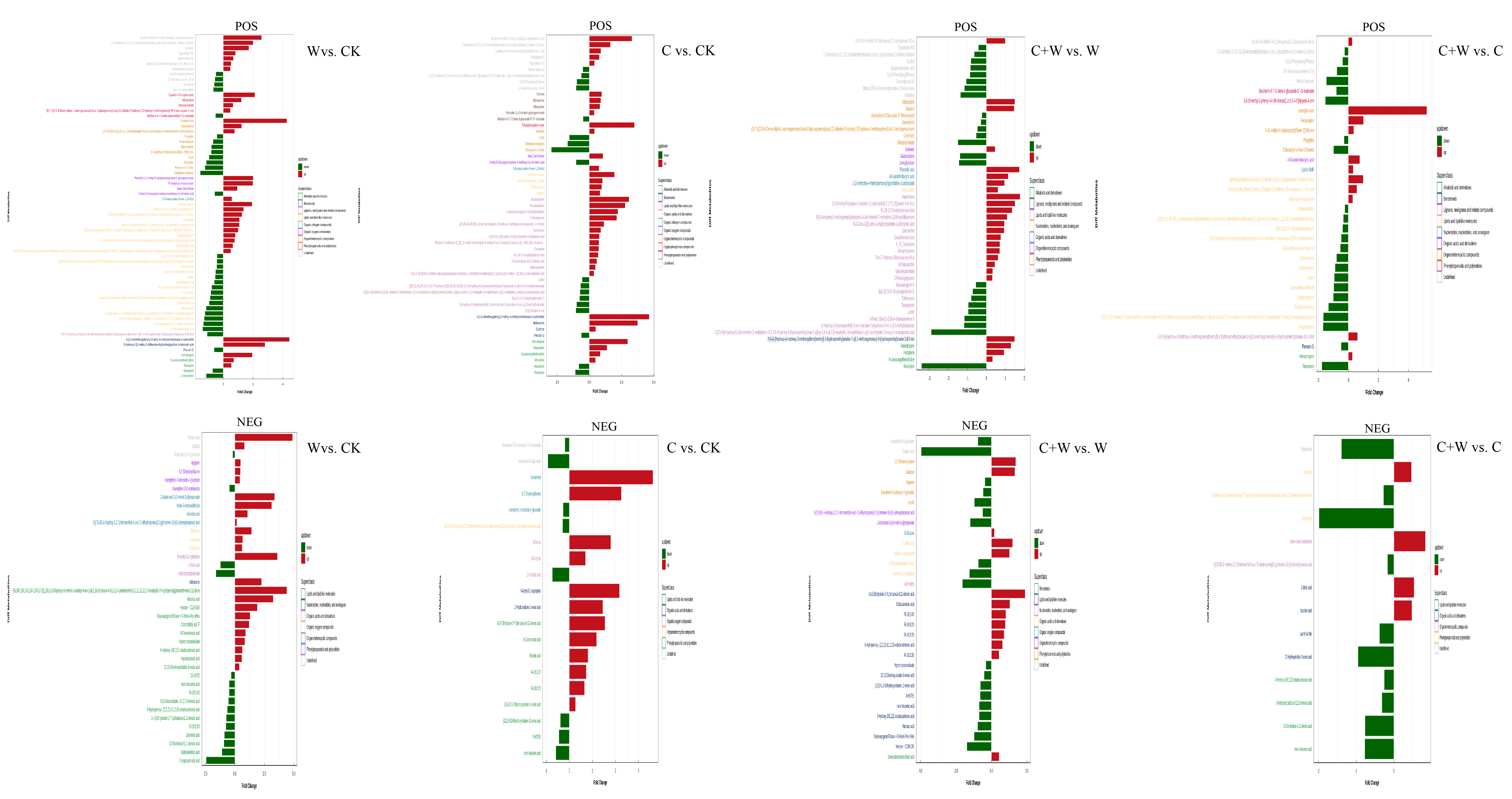

Supplement: Supplementary Figure S4 — Bar plot of the differential metabolites from each comparison group. [file Image4.jpeg]
